# IgG subclass deposition in diabetic nephropathy

**DOI:** 10.1186/s40001-022-00779-9

**Published:** 2022-08-11

**Authors:** Xuanli Tang, Feng Wan, Qin Zhu, Tian Ye, Xue Jiang, Haichun Yang

**Affiliations:** 1 Department of Nephrology (Key Laboratory of Zhejiang Province, Management of Kidney Disease), Hangzhou TCM Hospital Affiliated to Zhejiang Chinese Medical University, Hangzhou, 310007 China; 2 Department of Pathology, Microbiology, and Immunology, Vanderbilt University Medical Center, Nashville, TN USA

**Keywords:** IgG subclass, Diabetic nephropathy, Glomerular basement membrane, Tubular basement membrane

## Abstract

**Purpose:**

This study aimed to analyze the distribution of IgG subclass in diabetic nephropathy (DN) and its association with clinicopathological features.

**Methods:**

This is a single-center retrospective study enrolling 108 patients with biopsy-proven DN. Immunofluorescence and immunohistochemistry staining were applied, and clinicopathological features and renal outcomes were compared between patients with different patterns or categories of IgG subclass deposition.

**Results:**

Both IgG and its subclasses colocalized with collagen IV α5 on glomerular basement membrane (GBM) and some of tubular basement membrane (TBM). IgG1 and the Mixed type were two predominant types of deposition, no matter on GBM or TBM, and IgG1 showed a much higher deposition rate on GBM than that on TBM (*P* = 0.004). IgG subclass deposit on multi-location was more associated with a shorter duration of nephropathy and severer tubular interstitial injury (*P* < 0.05). The mixed type of IgG subclass deposit on GBM was merely associated with higher levels of proteinuria, whereas the deposition on TBM was more associated with higher levels of proteinuria, lower levels of albumin, more KIM-1 positive area, and thicker TBM (*P* < 0.05). Survival analysis revealed that none of the pattern or the category of IgG subclass deposit was a risk factor or a renal outcome indicator.

**Conclusions:**

IgG subclass was selectively deposited along GBM and/or TBM in DN, and the mixed type of IgG subclass deposition on TBM had more clinical significance than the isotype and that on GBM. IgG subclass deposition is merely a manifestation or a consequence rather than a cause in DN.

## Introduction

Diabetic nephropathy (DN) has a unique histological pattern, including glomerular basement membrane (GBM) thickening, mesangial expansion, and glomerulosclerosis, among others [1]. As a non-immunological related renal disease, the mechanism of IgG deposition along GBM and/or tubular basement membrane (TBM) in some DN cases is unclear [2]. This linear pattern cannot be detected by standard electron microscopy in DN, which is different from its deposition in immune complex-related glomerular diseases, such as membranous nephropathy (MN) [2]. Some hypotheses suggest that structural changes in the basement membrane lead to the entrapment of serum proteins, including albumin and IgG [3, 4]. One study demonstrated that up to 51.5% of the DN biopsies were IgG-positive, and the IgG intensity was associated with the progression of renal injury [2].

IgG has four subclasses (IgG1–G4); each subclass has a unique profile regarding half-life, antigen binding, immune complex formation, complement activation, and triggering of effector cells. Oxelius VA found that serum IgG2 and IgG3 levels declined, while IgG1 and IgG4 were relatively normal in juvenile diabetes mellitus (DM) cases [5]. Susanna M et al. found that serum IgG4 was selectively eliminated, and urinary IgG4 could be a helpful marker for preclinical stages of diabetic nephropathy [6]. Hemminger J et al. conducted a large retrospective study of IgG subclasses in 1084 routine renal biopsy cases regardless of the diagnosis and found that IgG4-dominant/codominant deposition with PLA2R-positive status was associated with primary MN, while IgG1 dominant/codominant with weak or absent IgG4 deposition was associated with autoimmune disease-related MN [7]. However, no IgG subclass in DN was included in this study. In 1984, Melvin T et al. studied IgG subclass in nine DN cases and found that only IgG4 had the same glomerular linear deposition as IgG [8]. This is difficult to explain considering the IgG profiles, since IgG4 has an anionic charge and the lowest serum concentration compared with the other subclasses, and GBM is anionically charged as well [6, 9]. Furthermore, the effect of tubular IgG deposition has not been studied. Therefore, we demonstrated the distribution of IgG subclass in 108 DN cases and analyzed its association with clinicopathological features and renal outcomes to explore the mechanism of IgG subclass deposition.

## Materials and methods

### Patients

Among 348 patients with type 2 DM and biopsy-proven DN between August 2017 and July 2021 at Hangzhou TCM Hospital Affiliated to Zhejiang Chinese Medical University, 225 cases showed an IgG linear pattern by immunofluorescence (IF). One hundred eight patients were enrolled according to the following inclusion criteria: (1) type 2 DM; (2) a diagnosis of DN proven by kidney biopsy; (3) IF showed IgG-positive status; and (4) Four IgG subclasses could be fully applied. The exclusion criteria were: (1) coexistence of nondiabetic renal diseases, such as MN, IgAN, or system diseases; (2) absence of glomeruli or global sclerosis in IF specimens; and (3) anti-GBM positive cases. The study was approved by the ethical committees of Hangzhou TCM Hospital Affiliated to Zhejiang Chinese Medical University (2020LH001).

### Clinicopathological characteristics and outcomes

The following clinical information was collected: age, gender, duration of diabetes, duration of nephropathy (start from the nephropathy symptoms such as lower extremities edema or soreness of waist till the diagnosis of DN), 24-h proteinuria, serum albumin, serum creatinine (Scr), and the estimated glomerular filtration rate (eGFR, calculated by the Chronic Kidney Disease Epidemiology Collaboration formula).

All specimens were processed for light microscopy (LM), IF, immunohistochemistry (IHC), and electron microscopy (EM). Patients were grouped according to the pattern of IgG subclass deposition (locating on GBM, TBM, or Both) and the category of deposition (the None group: no IgG subclass deposition; the Isotype group: deposition with only one IgG subclass; the Mixed group: deposition with more than two types of IgG subclass). Classification of DN and histological scoring were done according to the criteria reported by Tervaert et al. [1]. Interstitial fibrosis and tubular atrophy (IFTA, 0–3 score), as well as inflammation (0–2 score), arteria hyalinosis, and sclerosis (0–3 score), were scored according to methods described by the previous study [1]. Diagnosis, classification, and the score of these pathological findings were evaluated and confirmed by two renal pathologists. GBM thickness was measured by electron microscopy, and TBM thickness was measured by light microscopy according to studies reported by Haas and Tyagi I [10, 11]. Foot process effacement (FPE) was graded according to its severity (1–3 score) [12].

Patients were contacted by telephone, and the follow-up data included renal function (proteinuria and eGFR), dialysis, or kidney transplantation. The outcomes were progression to end stage renal disease (ESRD) or ≥ 50% decline in eGFR from baseline. ESRD was defined as eGFR < 15 mL/min/1.73 m2 or initiation of chronic renal replacement therapy.

### Immunofluorescence and immunohistochemistry staining

Frozen tissues were used for IgG (#F0202; 1:50, DAKO, Denmark) and IgG subclass (IgG1–IgG4, #F0767, #F4516, #F4641, #F9890, 1:50, Sigma-Aldrich, USA) staining by direct IF. Double staining of IgG or IgG subclass with anti-human collagen IV α5 (#C-452, 1:100, Cosmo corporation, Japan) by indirect IF (AF594 of Donkey anti-rat IgG as a secondary antibody, 1:100, Life Technologies, USA) was also performed. An Olympus BX53 fluorescence microscope (Japan) was used to analyze the IF slides.

IHC for kidney injury molecular-1 (KIM-1, #14,971, 1:200, Cell Signaling Technology, USA) and CD34 (ab81289, 1:100 titer, Abcam, Britain) was conducted using a Ventana BenchMark XT system. CD34 and KIM-1 positive statuses were analyzed by ImageJ and calculated as the percentage of positive area per glomeruli or cortex, respectively [13, 14].

### Statistical analysis

Statistical analysis was performed with SPSS 17.0. Normally distributed data were expressed as mean ± standard deviation, and non-normally distributed data were expressed as medians and interquartile ranges. A comparison of clinical and pathological characteristics among groups was assessed by *t* test or ANOVA for continuous variables and nonparametric tests for discontinuous variables. Categorical variables were expressed as percentages and comparisons among groups, which were evaluated by chi-square test or the Fisher’s exact test. The association between IgG subclass deposition and renal outcomes was evaluated using Cox proportional hazards models. The renal survival rates between patients with different patterns or categories of IgG subclass deposition were calculated using log-rank test and Kaplan–Meier analysis. A *P* value of less than 0.05 was considered significant.

## Results

### Demographics of clinicopathological features

**Fig. 1:**
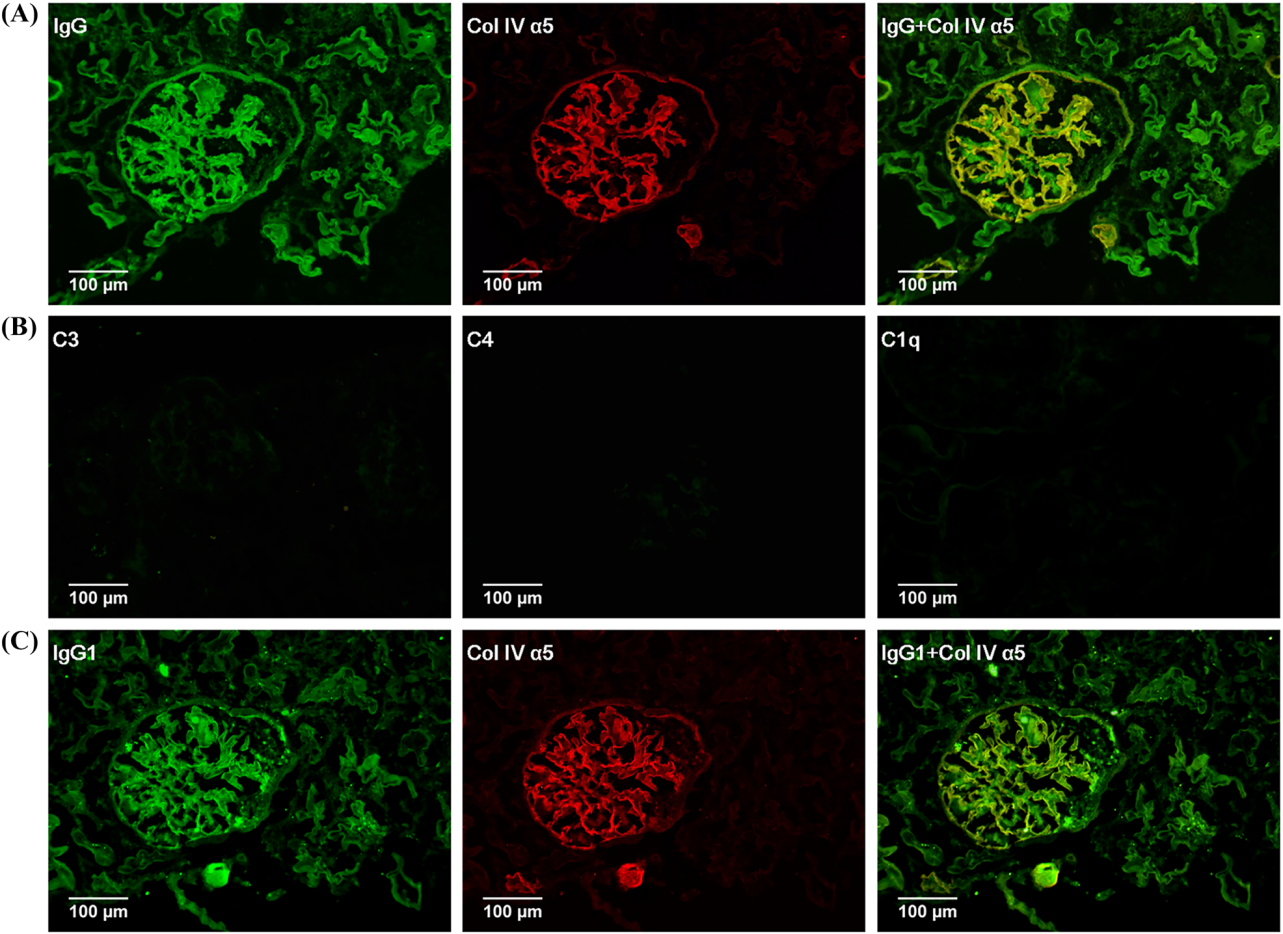
Linear deposition of IgG and IgG1 in diabetic nephropathy. **A** Glomerular linear IgG expression (Green), collagen IV α5 expression (Red), and colocalization of IgG and collagen IV α5 in glomeruli (IF, × 200); **B** negative C3, C4, and C1q in the same biopsy of figure A (IF, × 200); **C** glomerular linear IgG1 expression (Green), collagen IV α5 expression (Red), and colocalization of IgG1 and collagen IV α5 in glomeruli (IF, × 200)

At the time of renal biopsy of all 108 cases, the average age was 53.8 ± 9.3 years, and 77 cases comprised males. The median duration of type 2 DM and nephropathy was 96 (36, 120) and 2 (1, 12) months, respectively. Most of the patients enrolled in our study had nephrotic syndrome (the median urinary protein was 3.7 (1.4, 6.9) g/day and the median serum albumin was 30.9 (26.2, 37.2) g/L), with normal to a moderate reduction of renal function (median Scr 112.5 (75.0, 150.8) umol/L and median eGFR 65.0 (39.0, 102.0) ml/min per 1.73m^2^, respectively). Renal biopsy showed 65% of DN were in stage three. On average, the pathological features showed 23.1% glomerulosclerosis, moderate to severe FPE, and moderate IFTA. All cases had minor to moderate linear IgG expression along the GBM, similar to albumin, but only 56.5% of cases showed linear TBM deposition (Fig. 1A). IgG globally colocalized with collagen IV α5 along GBM but focally along TBM (Fig. 1A). All cases showed negative or trace complements (C3, C4, C1q) along GBM or TBM (Fig. 1B).

### Association between IgG subclass location and clinicopathological features

**Fig. 2:**
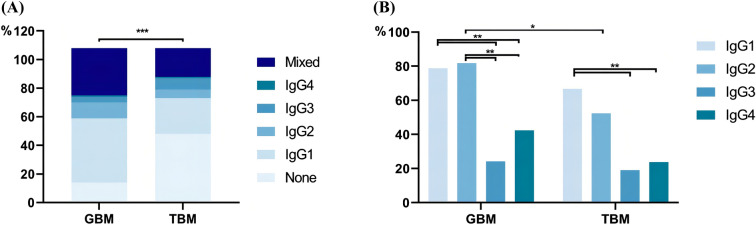
IgG subclass distribution on GBM and TBM. **A** Proportion of different types of IgG subclass deposit on GBM and TBM; **B** proportion of four IgG subclasses on GBM and TBM in the mixed group

One hundred cases showed one or more kinds of IgG subclass deposition along the GBM and/or TBM with a similar intensity of IgG, and the global linear deposition was colocalized with collagen IV α5 expression as well (Fig. 1C). IgG subclass showed higher frequency of deposition on GBM than that on TBM (87.0% vs 51.9%, *P* < 0.001) (Fig. 2). Among four kinds of IgG subclass deposit on GBM, cases of single IgG subclass accounted for 56.4%, with predominant IgG1 (41.7%), followed by IgG2 (10.2%), IgG3 (3.7%) and IgG4 (0.9%); in addition, mixed IgG subclass deposit accounted for 30.6%, and no deposit accounted for 13%. On the other hand, single IgG subclass deposit on TBM accounted for 37%, with predominant IgG1 (23.1%) as well, followed by IgG2 (5.6%), IgG3 (7.4%), and IgG4 (0.9%); in addition, mixed IgG subclass deposit accounted for 19.4%, and no deposit accounted for 43.5%. IgG1 showed a much higher deposition rate on GBM than that on TBM (*P* = 0.004) (Fig. 2). In the Mixed group, IgG1 and IgG2 had a higher deposition rate than IgG3 and IgG4 on GBM and/or TBM (*P* < 0.01), and IgG2 showed a higher deposition rate on GBM than that on TBM (*P* < 0.05) (Fig. 2).

Forty-four cases showed IgG subclass deposition along GBM only (the GBM-only group), whereas six were positive along TBM only (the TBM-only group). The remaining fifty cases had positive staining on both GBM and TBM (the Both group) (Table 1). None of the clinical features had significant differences between groups except that the duration of nephropathy was shorter in the TBM-only group than in the GBM-only group (*P* = 0.021). Pathological features, such as KIM-1 showed more positive area, and TBM exhibited thicker in the Both deposition group than in the GBM-only group (*P* < 0.05) (Table 1).

**Table 1:** Clinicopathological associations among different groups based on IgG subclass distribution

	None (*n* = 8)	GBM-only (*n* = 44)	TBM-only (*n* = 6)	Both (*n* = 50)	*P* Value
Male (%)	87.5	61.4	83.3	76.0	0.315
Age (year)	56.9 ± 10.0	51.4 ± 9.3	56.0 ± 8.4	55.1 ± 9.1	0.160
Duration of diabetes (mo)	102.0 (39.0, 144.0)	96.0 (36.0, 129.0)	60.0 (33.0, 129.0)	120.0 (39.0, 120.0)	0.804
Duration of nephropathy (mo)	**12.5 (1.0, 42.0)**	**4.5 (1.0, 21.0)**	**1.0 (1.0, 1.3)**	**1.0 (1.0, 6.0)**	**0.057**
Proteinuria(g/24 h)	3.2 (1.4, 4.9)	3.2 (1.1, 8.1)	3.4 (1.3, 5.4)	4.2 (1.5, 6.8)	0.727
Albumin (g/L)	32.6 ± 5.6	31.6 ± 8.2	29.8 ± 5.0	31.1 ± 6.6	0.896
Scr (μmol/L)	145.0 (75.0, 193.0)	95.0 (71.0, 140.0)	123.4 (65.5, 192.9)	118.0 (88.6, 146.5)	0.322
eGFR(ml/min/1.73m^2^)	44.5 (27.5, 117.0)	66.9 (42.0, 114.3)	100.5 (30.9, 106.1)	64.0 (37.0, 94.5)	0.442
DN stage I [n(%)]	1 (12.5)	3 (6.8)	1 (16.7)	3 (6.0)	0.456
DN stage II [n(%)]	3 (37.5)	7 (15.9)	1 (16.7)	15 (30.0)	0.279
DN stage III [n(%)]	3 (37.5)	32 (72.7)	4 (66.7)	31 (62.0)	0.254
DN stage IV [n(%)]	1 (12.5)	2 (4.5)	0 (0)	1 (2.0)	0.419
IgG intensity	1.0 ± 0.0	1.3 ± 0.6	1.2 ± 0.4	1.2 ± 0.5	0.533
C3 intensity	0.0 (0.0, 1.0)	0.0 (0.0, 0.0)	0.0 (0.0, 0.6)	0.0 (0.0, 0.5)	0.693
GS (%)	23.2 (11.5, 41.6)	16.7 (7.3, 30.6)	13.4 (4.7, 24.8)	19.8 (11.1, 38.3)	0.480
CD34 + area (%)	23.2 ± 8.1	25.3 ± 8.5	25.1 ± 8.1	23.6 ± 8.0	0.750
GBM thickness (nm)	715.0 ± 158.3	770.8 ± 174.7	709.2 ± 109.3	734.8 ± 195.3	0.743
FPE	2.6 ± 0.5	2.6 ± 0.6	2.8 ± 0.5	2.7 ± 0.7	0.918
IFTA score	1.8 ± 0.7	2.0 ± 0.6	2.2 ± 0.8	2.1 ± 0.7	0.607
KIM-1 + area (%)	**20.0 (15.0, 37.5)**	**25.0 (10.0, 37.5)**	**20.0 (17.5, 28.8)**	**30.0 (20.0, 42.5)**	**0.061**
TBM thickness (nm)	**1207.3 ± 316.4**	**1080 ± 287.1**	**1250 ± 273.1**	**1345 ± 476.9**	**0.016**
Inter-infla score	2.0 (1.0, 2.0)	2.0 (2.0, 2.0)	2.0 (1.5, 3.0)	2.0 (2.0, 2.0)	0.559
Hyalinosis score	1.9 ± 0.4	1.8 ± 0.5	1.7 ± 0.7	1.7 ± 0.5	0.835
A-sclerosis score	1.0 (0.0, 1.0)	1.0 (0.0, 1.0)	1.0 (0.8, 1.0)	1.0 (0.0, 1.0)	0.926

Data with significant differences are highlighted in bold

Scr, serum creatinine; eGFR, estimated glomerular filtration rate; DN, diabetic nephropathy; GS, Glomerular sclerosis; FPE, foot process effacement; IFTA, interstitial fibrosis and tubular atrophy; KIM-1, kidney injury molecular-1; TBM, tubular basement membrane; inter-infla, interstitial inflammation; A-sclerosis score, Arteriosclerosis score

### Association between IgG subclass deposition on GBM and clinicopathological features

**Fig. 3:**
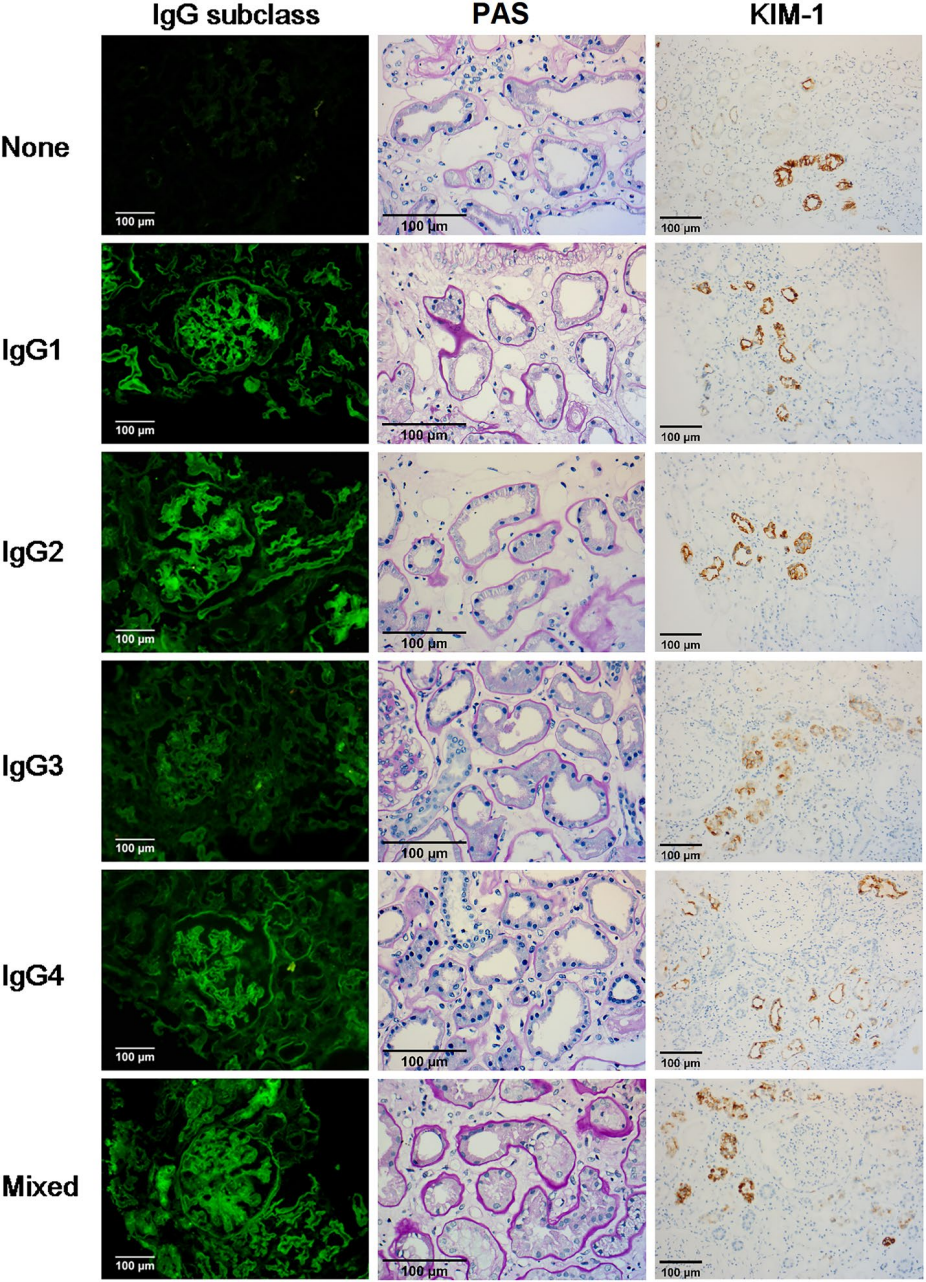
Types of IgG subclass and their pathological features. Thicker TBM and more KIM-1 positive area were observed in the isotype and the mixed group than in the none group. (IgG subclass: IF, × 200; PAS: × 200; KIM-1: IHC, × 200)

Ninety-four cases showed IgG subclass deposit on GBM, including 61 cases from the Isotype group and 33 cases from the Mixed group (Table 2). None of the clinicopathological data showed a significant difference, except that proteinuria exhibited much higher in the Mixed group than that in the Isotype group and the None group (*P* = 0.018) (Fig. 3).

**Table 2:** Clinicopathological findings of IgG subclass deposit on GBM

	None (*n* = 14)	Isotype (*n* = 61)	Mixed (*n* = 33)	*P* Value
Male (%)	85.7	67.2	72.7	0.365
Age (year)	56.5 ± 9.0	53.3 ± 8.8	53.5 ± 10.4	0.497
Duration of diabetes (mo)	90.0 (36.0, 129.0)	96.0 (36.0, 120.0)	120.0 (45.0, 141.1)	0.857
Duration of nephropathy (mo)	1.0 (1.0, 24.0)	2.0 (1.0, 12.0)	5.5 (1.0, 8.0)	0.465
Proteinuria(g/24 h)	**3.3 (1.1, 4.8)**	**3.2 (1.3, 6.0)**	**6.5 (1.7, 9.1)**	**0.047**
Albumin (g/L)	31.4 ± 5.4	32.1 ± 7.4	29.8 ± 7.2	0.337
Scr(μmol/L)	145.0 (71.9, 182.0)	117.0 (72.8, 154.5)	103.0 (82.9, 132.5)	0.346
eGFR (ml/min/1.73m^2^)	46.9 (29.0, 109.1)	64.0 (36.7, 112.0)	65.0 (52.0, 96.1)	0.838
DN stage I [n(%)]	2 (14.3)	5 (8.2)	1 (3.0)	0.272
DN stage II [n(%)]	4 (28.6)	14 (23.0)	8 (24.2)	0.857
DN stage III [n(%)]	7 (50.0)	40 (65.6)	23 (69.7)	0.429
DN stage IV [n(%)]	1 (7.1)	2 (3.3)	1 (3)	0.598
IgG intensity	1.0 (1.0, 1.0)	1.0 (1.0, 1.0)	1.0 (1.0, 1.5)	0.384
C3 intensity	0.0 (0.0, 1.0)	0.0 (0.0, 0.0)	0.0 (0.0, 0.5)	0.467
GS (%)	14.9 (9.9, 36.1)	23.2 (10.0, 40.0)	16.7 (5.1, 25.6)	0.360
CD34 + area (%)	24.0 ± 7.8	23.2 ± 7.6	26.7 ± 9.0	0.134
GBM thickness (nm)	711.8 ± 126.5	776.9 ± 183.3	713.2 ± 184.9	0.247
FPE	3.0 (2.0, 3.0)	3.0 (2.0, 3.0)	3.0 (2.5, 3.0)	0.707
IFTA score	1.9 ± 0.8	2.1 ± 0.6	2.0 ± 0.7	0.816
KIM-1 + area (%)	20.0 (15.0, 32.5)	30.0 (15.0, 40.0)	30.0 (16.3, 47.5)	0.500
TBM thickness (nm)	1225.8 ± 288.3	1215.1 ± 354.2	1234.2 ± 524.3	0.976
Inter-infla score	2.0 (1.0, 2.5)	2.0 (2.0, 2.0)	2.0 (2.0, 2.0)	0.850
Hyalinosis score	1.8 ± 0.5	1.7 ± 0.5	1.8 ± 0.4	0.507
A-sclerosis score	1.0 (0.3, 1.0)	1.0 (0.0, 1.0)	1.0 (0.0, 1.0)	0.948

Data with significant differences are highlighted in bold

GBM, glomerular basement membrane; Scr, serum creatinine; eGFR, estimated glomerular filtration rate; DN, diabetic nephropathy; GS, Glomerular sclerosis; FPE, foot process effacement; IFTA, interstitial fibrosis and tubular atrophy; KIM-1, kidney injury molecular-1; TBM, tubular basement membrane; inter-infla, interstitial inflammation; A-sclerosis score, Arteriosclerosis score

### Association between IgG subclass deposition on TBM and clinicopathological features

TBM showed a much lower frequency (56.5%) of IgG subclass deposit than GBM, including 37% from the Isotype group and 19.5% from the Mixed group (Table 3). The clinicopathological data showed a shorter duration of nephropathy, more KIM-1 positive area and thicker TBM in the Isotype and the Mixed group than in the None group (*P* < 0.05); meanwhile, higher levels of proteinuria and lower levels of serum albumin exhibited in the Mixed group than in the Isotype group and the None group (*P* < 0.05).

**Table 3:** Clinicopathological findings of IgG subclass deposit on TBM

	None (*n* = 47)	Isotype (*n* = 40)	Mixed (*n* = 21)	*P* Value
Male (%)	68.1	70.0	81.0	0.542
Age (year)	52.7 ± 9.7	54.9 ± 9.0	54.0 ± 9.2	0.556
Duration of diabetes (mo)	96.0 (45.0, 156.0)	96.0 (27.0, 120.0)	114.0 (36.0, 120.0)	0.816
Duration of nephropathy (mo)	**6.0 (1.0, 24.0)**	**1.0 (1.0, 6.0)**	**2.0 (1.0, 6.0)**	**0.023**
Proteinuria(g/24 h)	**2.9 (0.6, 7.0)**	**3.8 (0.9, 6.0)**	**6.6 (3.3, 8.2)**	**0.108**
Albumin (g/L)	**32.4 ± 8.0**	**32.0 ± 6.5**	**27.8 ± 5.0**	**0.035**
Scr(μmol/L)	101.0 (71.5, 145.5)	118.0 (82.4, 153.8)	110.0 (76.0, 164.4)	0.482
eGFR (ml/min/1.73m^2^)	65.0 (42.0, 114.0)	65.5 (35.6, 97.9)	61.0 (27.0, 104.3)	0.569
DN stage I [n(%)]	4 (8.5)	4 (10.0)	0 (0)	0.450
DN stage II [n(%)]	11 (23.4)	10 (25.0)	5 (23.8)	1.000
DN stage III [n(%)]	29 (61.7)	25 (62.5)	16 (76.2)	0.523
DN stage IV [n(%)]	3 (6.4)	1 (2.5)	0 (0)	0.530
IgG intensity	1.3 ± 0.6	1.2 ± 0.4	1.2 ± 0.4	0.538
C3 intensity	0.0 (0.0, 0.5)	0.0 (0.0, 0.0)	0.0 (0.0, 0.5)	0.880
GS (%)	17.0 (10.0, 32.4)	24.4 (10.0, 40.0)	14.2 (8.0, 24.5)	0.398
CD34 + area (%)	25.4 ± 9.2	22.8 ± 7.3	25.0 ± 7.0	0.309
GBM thickness (nm)	754.7 ± 180.8	747.3 ± 165.8	731.3 ± 203.3	0.899
FPE	2.6 ± 0.6	2.7 ± 0.7	2.8 ± 0.4	0.524
IFTA score	2.0 ± 0.6	2.1 ± 0.6	1.9 ± 0.8	0.410
KIM-1 + area (%)	**20.0 (10.0, 30.0)**	**30.0 (20.0, 40.0)**	**30.0 (20.0, 50.0)**	**0.008**
TBM thickness (nm)	**1067.9 ± 282.4**	**1320.5 ± 365.4**	**1381.0 ± 570.1**	**0.001**
Inter-infla score	2.0 (2.0, 2.0)	2.0 (2.0, 3.0)	2.0 (1.0, 2.5)	0.484
Hyalinosis score	1.8 ± 0.4	1.7 ± 0.5	1.7 ± 0.5	0.331
A-sclerosis score	1.0 (0.0, 1.0)	1.0 (0.0, 1.0)	1.0 (0.5, 1.0)	0.519

TBM, tubular basement membrane; Scr, serum creatinine; eGFR, estimated glomerular filtration rate; DN, diabetic nephropathy; GS, Glomerular sclerosis; FPE, foot process effacement; IFTA, interstitial fibrosis and tubular atrophy; KIM-1, kidney injury molecular-1; TBM, tubular basement membrane; Inter-infla, interstitial inflammation; A-sclerosis score, Arteriosclerosis score

### Survival analysis

The survival analysis presented that none of any IgG subclass or the pattern of deposition was a risk factor for renal outcomes by Cox regression analysis, as well as no significant survival difference was found among different deposition groups by log-rank test.

## Discussion

In our study, 64.7% of the DN cases showed linear IgG staining by IF, lower than that reported by Mise K but higher than that of Zhang J [2, 15]. Unlike other immune complex-mediated glomerular nephropathy, such as membranous nephropathy, both IgG and albumin showed linear deposition but without dense deposits along GBM and TBM, and complements were negative. Furthermore, eluates from DN kidneys did not contain anti-GBM antibody as reported [16]. Those manifestations suggest that this kind of IgG deposition might occur more frequently as a manifestation or a consequence of renal injury [3, 4, 17].

IgG subclass is a useful diagnostic tool for several renal diseases, including MN, heavy and light chain deposition disease, proliferative glomerulonephritis with polyclonal IgG deposition, etc. [7]. The different locations of IgG subclass deposition are determined by both immunoglobulin profiles and the local environment. While the molecular size is similar among the four subclasses of IgG, the charge and serum concentration decreased in the order of IgG1, IgG2, IgG3, and IgG4 [6, 9, 18, 19]. Owing to the anionic charge feature of GBM, the affinity of IgG subclass to GBM should be higher for IgG1 and IgG2 than that of IgG4 [20, 21]. Zhang et al. reported that IgG1 deposit along the GBM and TBM tended to be prevalent in IgG positive patients with DN. However, no correlation was found between the IgG subclass distribution along the GBM and clinicopathological data or renal prognosis of DN patients [15]. In our study, IgG1 and the mixed type with predominant IgG1 and IgG2 were two major types of IgG subclass deposition, no matter on GBM or TBM, which was partly consistent with Zhang et al. [15]. We suspect the cationic charge of IgG1 plays the main role of deposition.

The deposition pattern of IgG subclass showed that GBM-only and multi-location deposits were predominant in DN. IgG subclass deposits on multi-location was more associated with a shorter duration of nephropathy and severer tubular interstitial injury, which shows multi-location deposit of IgG subclass indicates rapid progression of DN to some extent, though there was no significant findings of the association between the location and renal outcomes after survival analysis. Furthermore, the deposition of IgG subclass on TBM was diffuse, and the location of deposition did not show any preference of proximal or distal tubules; meanwhile, both preserved and injured tubules exhibited the same intensity of IgG subclass deposition, which suggests the mechanism of TBM deposition might be not only due to the re-absorption of proximal tubules but also the leakage of peritubular capillaries around [22].

To explore the category of IgG subclass deposit on GBM, we divided them into three groups according to the number of types of subclass deposits. Out of our expectations, no significant difference except higher levels of proteinuria was observed in the Mixed group than in the Isotype group. We suspect it might be due to the complexity of the structure of GBM (probably charge property, pore size, and slit diagram) and the crosstalk between endothelial cells and podocytes [17–21, 23, 24]. However, the TBM deposition of IgG subclass seemed to have more clinicopathological significance, which had always been overlooked by pathologists. In our study, some of the clinical and pathological data showed that more kinds of IgG subclass deposition were associated with severer injuries of both glomeruli and tubules, which reminds pathologists and nephrologists that more attention should be paid to the location of TBM deposition and the mixed type of IgG subclass rather than that of GBM deposition and the isotype deposition.

It’s reported that the glomerular IgG deposit emerged as an independent risk factor for renal clinical outcomes [15]. However, the survival analysis in our study revealed that neither the type of IgG subclass nor the deposition pattern was a risk factor or to be associated with renal outcome. We suspect the deposition of IgG subclass is merely a manifestation or a consequence, as we speculated from the pathological features above, which would not have an impact on the prognosis of DN. The limitation of this study is the single-center experience, which needs multi-center confirmation.

In summary, the location and the category of IgG subclass deposit are probably determined by their profiles and the severity of glomerular/tubular injury. The mixed type of IgG subclass deposition on TBM had more clinical significance than the isotype and that on GBM. IgG subclass deposition is merely a manifestation or a consequence rather than a cause in DN.

## Data Availability

The data sets used or analysed during the current study are available from the corresponding author on reasonable request.

## Abbreviations

DN: Diabetic nephropathy
GBM: Glomerular basement membrane
TBM: Tubular basement membrane
MN: Membranous nephropathy
DM: Diabetes mellitus
IF: Immunofluorescence
Scr: Serum creatinine
eGFR: Estimated glomerular filtration rate
LM: Light microscopy
IHC: Immunohistochemistry
EM: Electron microscopy
IFTA: Interstitial fibrosis and tubular atrophy
FPE: Foot process effacement
ESRD: End stage renal disease
KIM-1: Kidney injury molecular-1
